# Effects of Dietary Valine Chelated Zinc Supplementation on Growth Performance, Antioxidant Capacity, Immunity, and Intestine Health in Weaned Piglets

**DOI:** 10.1007/s12011-023-03870-2

**Published:** 2023-09-20

**Authors:** Tuan Zhang, Nan Zhang, Shuyu Peng, Yawei Zhang, Huakai Wang, Shiyu Huang, Min Zhu, Yongxi Ma

**Affiliations:** 1 State Key Laboratory of Animal Nutrition, College of Animal Science and Technology, China Agricultural University, Beijing, 100193 China; 2 Changsha Xinjia Bio-Engineeriong Co., Ltd, Changsha, China

**Keywords:** Valine chelated zinc, Antioxidant capacity, Immunity function, Intestinal health, Piglets

## Abstract

This study was conducted to investigate the effects of dietary valine chelated zinc (ZnVal) supplementation on growth performance, antioxidant capacity, immunity, and intestine health in weaned piglets. A total of 240 healthy 35-day-old weaned piglets (Duroc × Landrace × Yorkshire, average weight 10.70 ± 0.14 kg) were randomly divided into five groups with six replicate pens and eight piglets per pen. Dietary treatments were a corn–soybean meal basal diet supplemented with 0, 25, 50, 75, and 100 mg/kg ZnVal, respectively. The experiment lasted for 28 days. Results showed that average daily gain (ADG) was increased (*P* < 0.05) by ZnVal with 75–100 mg/kg supplementation on days 15–28 and with 50–100 mg/kg supplementation on days 1–28. Supplementation of 25–100 mg/kg ZnVal reduced (*P* < 0.01) the diarrhea rate of weaned piglets on days 1 to 14 and 1 to 28. Dietary supplementation with 25–100 mg/kg ZnVal increased (*P* < 0.05) copper/zinc-superoxide dismutase (Cu/Zn-SOD) and decreased malonaldehyde (MDA) activities in the serum on day 14 and 28. Supplementation of 25–100 mg/kg ZnVal increased (*P* < 0.05) glutathione peroxidase (GSH-Px) activity in serum on day 14. Additionally, the supplementation of 75 mg/kg ZnVal significantly increased the activity of superoxide dismutase (SOD) and Cu/Zn-SOD in the liver (*P <* 0.05). Furthermore, the supplementation of 25–100 mg/kg ZnVal significantly increased the total antioxidant capacity (T-AOC) in the liver (*P <* 0.05). Higher (*P* < 0.05) concentrations of IgG in the serum were measured from piglets supplemented with 75–100 mg/kg ZnVal on day 14 and dietary supplementation with 25–100 mg/kg ZnVal increased the level of immunoglobulin G (IgG) in serum on day 28 (*P* < 0.05). In addition, higher (*P* < 0.05) concentrations of immunoglobulin A (IgA) in the duodenum and ileum were measured from piglets supplemented with 75 mg/kg ZnVal and the supplementation of 25–100 mg/kg ZnVal also showed a higher (*P* < 0.05) concentration of immunoglobulin G (IgG) in duodenum. Supplementation of 50–100 mg/kg ZnVal increased the villus height and villus height/crypt depth of jejunum (*P* < 0.05). Moreover, dietary supplementation with 75–100 mg/kg ZnVal showed a higher (*P* < 0.05) concentration of zinc in the liver and supplementation of 50–100 mg/kg ZnVal increased (*P* < 0.05) the concentration of zinc in the heart, spleen, and kidney. In conclusion, the present research showed that supplementation of ZnVal improves growth performance by increasing antioxidant capacity and immunity and regulating intestinal morphology and the optimal inclusion level of ZnVal was 65~80 mg/kg.

## Introduction

Zinc is an integral part of more than 200 enzymes in mammals, directly involved in various physiological processes, such as DNA and protein synthesis, proliferation, differentiation, and apoptosis in the entire life [1–4]. It plays a role in many enzymes or acts as a cofactor of many enzymes, and a deficiency of zinc reduces cellular immunity [5, 6]. In recent years, zinc sources have experienced the development process from inorganic zinc to organic zinc form, including amino acid chelated zinc [7]. Among them, several studies have shown that amino acid chelated zinc has higher biological efficacy, utilization rate, and better stability, which could improve growth performance and reduce environmental pollution [7, 8].

**Scheme 1:**
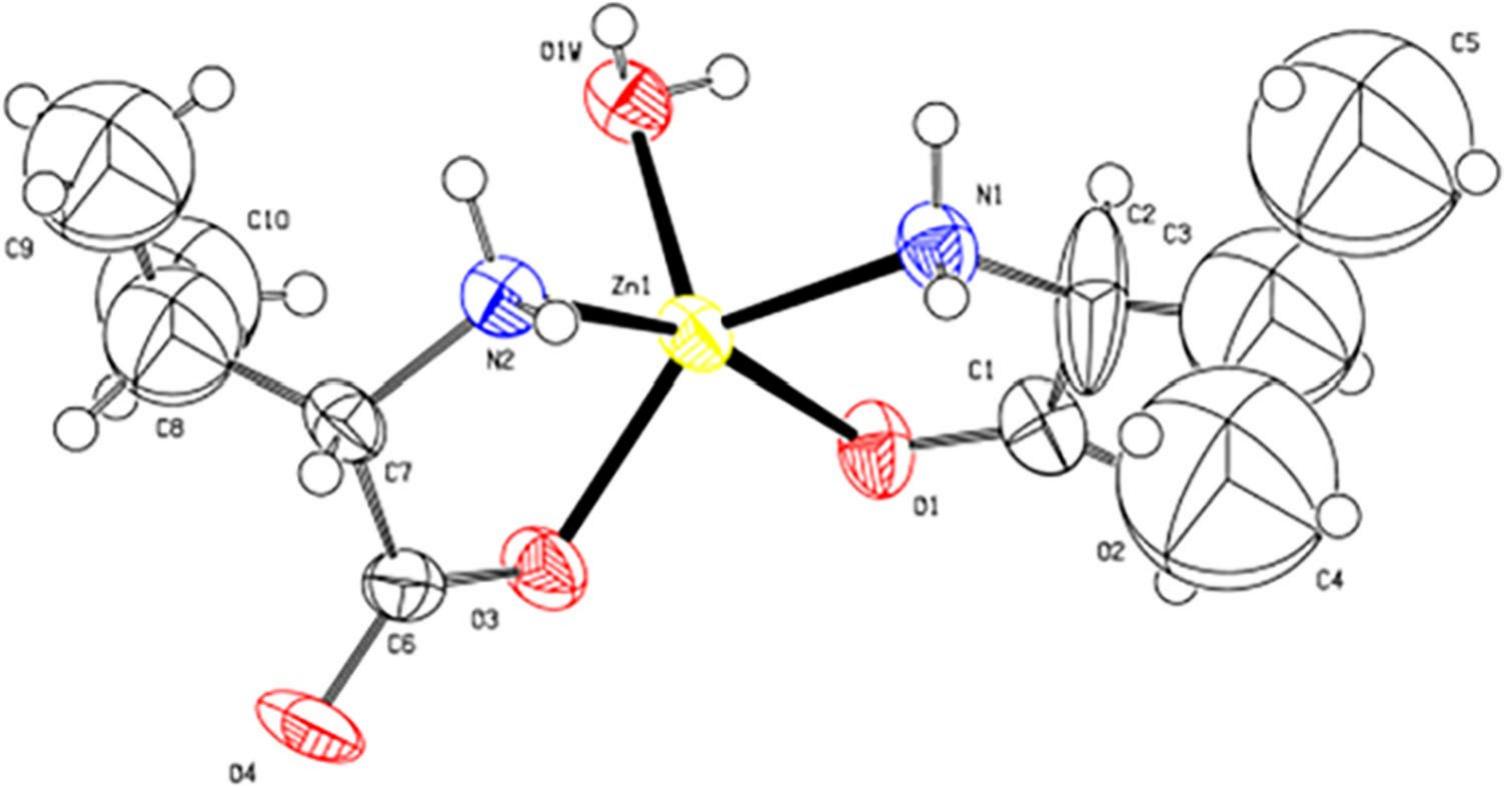
The chemical structural formula of ZnVal. Note: This picture was provided by Changsha Xinjia Bio-Engineeriong Co., Ltd

Valine chelated zinc [Zn(C_5_H_10_NO_2_)_2_·2H_2_O] is a five-membered ring structure consisting of valine and zinc ion in a 2:1 molar ratio (Scheme 1). It is reported that amino acid chelated zinc can facilitate the absorption of trace minerals effectively as it can be absorbed directly as a whole through the cell membrane into the plasma, while inorganic zinc must be chelated with amino acids or other substances, such as coenzyme, to be absorbed [8–10]. In addition, amino acid chelated zinc is reported to protect zinc from reactions with phytates, also leading to higher bioavailability [11]. In addition, Li et al. [9] showed that 60 mg/kg ZnMet increased the T-AOC and GSH-Px activity in serum and the T-AOC, Cu/Zn-SOD, and GSH-Px activity in the liver of laying hens. Zhu et al. [12] found that the zinc glycinate group had higher GSH-Px and lower MDA concentrations than the broilers in the ZnSO_4_ groups. Besides, it was indicated that diets supplemented with lower levels of Zn-threonine and Zn-methionine can maintain egg production performance, improve egg quality, enrich eggs, and increase bioavailability [13]. Previous studies have shown that amino acid chelated zinc could improve antioxidant capacity and immunity in hens [9, 12]. The effects of ZnVal on piglets are poorly known. Hence, the objective of this study was to evaluate the effects of dietary ZnVal supplementation on growth performance, antioxidant capacity, immunity, and intestine health in weaned piglets.

## Materials and Methods

All animal experiments were approved by the animal ethics committee of China Agricultural University (Beijing, China; NO. AW11093202-1-1). All animal experiments were performed at the FengNing Swine Research Unit of China Agricultural University (Chengdejiuyun Agricultural and Livestock Co., Ltd., Hebei, China). The main active components of the ZnVal used in the study are zinc (19.31%) and valine (68.11%) and provided by Changsha Xinjia Bio-Engineeriong Co., Ltd (Changsha, China).

### Animals and Experimental Design

A total of 240 (Duroc × Landrace × Yorkshire) weaned piglets with an average initial body weight (BW) of 10.70 ± 0.14 kg were randomly allotted to five treatments with six replicates (8 piglets per replicate pen) per treatment based on BW and sex. The corn–soybean basal diets were formulated to meet the recommendations for 8 to 25 kg BW piglets by Nutrient Requirements of Swine (GB/T 39235-2020) for 8- to 25-kg BW piglets and are shown in Table 1 [14]. The dietary treatments included a corn–soybean basal diet (CON, without zinc supplementation) and the basal diets added with 25, 50, 75, and 100 mg/kg ZnVal.. Crystalline valine was added as needed to each diet to maintain a consistent dietary valine content. The piglets were housed in pens with relatively constant temperature (26~28°C) and humidity (60~70%) and were given access to feed and water ad libitum throughout the experiment period (28 days).

**Table 1:** Ingredients composition of the basal diets (%, as-fed basis)

Ingredients	ZnVal (mg/kg)				
	0	25	50	75	100
Corn	66.9	66.9	66.9	66.9	66.9
Soybean meal	24.4	24.4	24.4	24.4	24.4
Soybean oil	3.0	3.0	3.0	3.0	3.0
Fish meal	2.0	2.0	2.0	2.0	2.0
Dicalcium phosphate	1.1	1.1	1.1	1.1	1.1
Limestone	0.7	0.7	0.7	0.7	0.7
L-Lysine, 78%	0.6	0.6	0.6	0.6	0.6
Methionine, 98.5%	0.1	0.1	0.1	0.1	0.1
Tryptophan, 98%	0.2	0.2	0.2	0.2	0.2
Tryptophan, 98%	0.1	0.1	0.1	0.1	0.1
NaCl	0.3	0.3	0.3	0.3	0.3
Choline chloride, 50%	0.1	0.1	0.1	0.1	0.1
Premix^1^	0.5	0.5	0.5	0.5	0.5
Nutrient levels^2^					
Digestible energy (MJ/kg)	14.39	14.39	14.39	14.39	14.39
Crude protein, %	18.55	18.68	18.55	18.65	18.62
Lysine, %	1.38	1.37	1.37	1.36	1.38
Methionine, %	0.41	0.41	0.42	0.43	0.41
Threonine, %	0.86	0.86	0.86	0.86	0.87
Tryptophan, %	0.30	0.30	0.29	0.30	0.29
Valine, %	0.87	0.89	0.89	0.87	0.86
Zinc, mg/kg	22.96	45.66	71.08	96.04	121.54
Total calcium, %	0.64	0.66	0.61	0.62	0.65
Total phosphorus, %	0.47	0.48	0.47	0.47	0.50

^1^The premix provided the following (per kilogram of compound feed): vitamin A, 12,000 IU; vitamin D_3_, 2000 IU; vitamin E, 24 IU; vitamin K_3_, 2.0 m; vitamin B_1_, 2.0 mg; vitamin B_6_, 3.0 mg; vitamin B_12_, 24 μg; riboflavin, 6.0 mg; nicotinic acid, 30 mg; d-pantothenic acid, 20 mg; folic acid, 3.6 mg; biotin, 0.1 mg; choline chloride, 400 mg; Fe, 96 mg; Cu, 8.0 mg; Mn, 40 mg; I, 0.56 mg; Se, 0.4 mg

^2^Nutrient levels are calculated according to GB/T 39235-2020; all of the data are analyzed except for digestible energy

### Growth Performance and Diarrhea Rate

At the beginning and end of the experiment, all piglets were weighed individually, and residual feed was collected and weighed for each pen; ADG, ADFI, and FCR were calculated. Occurrences of diarrhea were recorded every day at 09:00 using the scoring system described by Yang et al. [15]. The scoring system was applied to determine the rate of diarrhea as follows: 0 = hard feces; 1 = slightly soft feces; 2 = soft, partially formed feces; 3 = loose, semiliquid feces; 4 = watery, mucous-like feces. When the average score was over 2 for 2 consecutive days, piglets were identified as having diarrhea. The diarrhea rate was calculated according to the following equation [16]:

$$\textrm{Diarrhea}\ \textrm{rate}\ \left(\%\right)=\textrm{the}\ \textrm{number}\ \textrm{of}\ \textrm{diarrhea}\ \textrm{piglets}\times \textrm{diarrhea}\ \textrm{days}/\left(\textrm{the}\ \textrm{total}\ \textrm{number}\ \textrm{of}\ \textrm{piglets}\times \textrm{experiment}\ \textrm{days}\right)\times 100$$

### Sample Collection

During this experiment, approximately 2.0-kg representative feed samples were taken from each treatment group. On the morning of days 15 and 28, one piglet in each pen from CON and ZnVal groups was selected for collection of a blood sample via jugular vena puncture with vacutainer tubes (Becton Dickinson Vacutainer Systems, Franklin Lakes, NJ, USA). Blood samples were centrifuged at 3000 × *g* for 10 min to obtain the serum and stored at −20°C until analysis.

On day 28, 6 piglets from each treatment group close to the median body weight were selected and euthanized. The small intestine was removed from the abdominal cavity, and divided into three parts: duodenum, jejunum, and ileum according to the method described by Peng et al [15]. Intestinal samples from the middle regions of the duodenum, jejunum, and ileum were collected after removing its contents and washed with saline. Then, the intestinal samples were preserved in 4% paraformaldehyde for 24 h for morphological examination. The heart, liver, spleen, and kidney were stored at −20°C until analysis. Moreover, an approximately 2 cm length of the duodenum, ileum, and jejunum were collected and stored at –80°C for immunoglobulin analysis.

### Sample Analysis

Association of Official Analytical Chemists [17] methods were used when testing nutrients in the diets including total phosphorus (AOAC method 995.11), calcium (AOACmethod 927.02), crude protein (CP) (AOAC method 988.05), and amino acids (AOAC method 994.12). Before acid hydrolysis with 6 M HCl, performic acid oxidation was completed to determine dietary methionine content. Zinc content in the diets and tissues was analyzed using an inductively coupled plasma (LCP) atomic absorption spectrometer (NOVAA350, Jena, Germany).

Collect about 0.2 g of liver tissue sample and add 900 times the volume of saline. Homogenize and centrifuge at 3000 × *g* for 10 min in order to obtain the supernatant for determining liver antioxidant enzymes. The activity of T-AOC, GSH-Px, and SOD and MDA concentration in serum and liver were measured using biochemical methods following the manufacturer’s instructions provided for each reagent kit (Nanjing Jiancheng Bioengineering Institute, Nanjing, China). Briefly, the content of MDA was determined by the thiobarbituric acid reaction. The activities of T-AOC, GSH-Px, and SOD were assayed using the xanthine oxidase, the dithiodinitrobenzoic acid colorimetric, and the ammonium molybdate method, respectively.

The activity of Cu/Zn-SOD in serum and liver and the levels of IgA, IgM, and IgG in serum, duodenum, jejunum, and ileum were measured using ELISA kits following the manufacturer’s instructions (Nanjing Jiancheng Bioengineering Institute, Nanjing, China).

The fixed intestinal specimens preserved in 4% paraformaldehyde were dehydrated, cleared, and embedded in paraffin wax using standard paraffin embedding techniques. The samples were sectioned at 5-μm thickness and installed on glass slides. Then, the paraffin sections were dewaxed and stained with hematoxylin and eosin (H&E). Villus height and crypt depth were measured under a microscope with 40×-combined magnification using image processing and an analysis system (version 1; Leica Imaging Systems Ltd., Cam-bridge, UK). At least 12 well-oriented intact villi and the associated crypt depth of each section were identified and measured. The ratio of villus height to crypt depth was calculated.

### Statistical Analysis

Data from this study were analyzed by one-way analysis of variance with the GLM procedure of SAS 9.4 (SAS Institute Inc., Cary, NC, USA). The pen was used as the experimental unit for growth performance, and individual pig was the experimental unit for antioxidant capacity, immunity, and intestinal morphology. The UNIVARIATE procedure of SAS 9.4 (SAS Institute, Cary, NC, USA) was used to verify the normality of all data except diarrhea rate. Duncan’s multiple comparison test was used to determine the difference among treatments. The linear and quadratic effects of ZnVal levels were evaluated using orthogonal polynomial contrasts. A quadratic regression fitting curve model was performed using GraphPad Prism 9 to evaluate the optimal level of ZnVal in the diets. Difference was deemed significant when the *P* value was less than 0.05 and 0.05 ≤ *P* < 0.10 was considered a significant trend.

## Results

### Growth Performance

The effects of dietary ZnVal supplementation on growth performance and diarrhea rate are shown in Table 2. Dietary ZnVal supplementation did not affect ADFI and FCR on days 1–14, 15–28, and 1–28; however, it tended to increase ADG on days 1 to 14 (*P* = 0.09) and ADG was increased by ZnVal with 75–100 mg/kg supplementation on days 15–28 and with 50–100 mg/kg supplementation on days 1–28. According to the quadratic fit model of dietary ZnVal supplementation level, the optimal supplemental level of ZnVal for piglets based on ADG (*Y* = −8.344 × 10^−3^*X*^2^ + 1.255*X* + 439.6, *R*^2^ = 0.97) from days 1–28 was 75.2 mg/kg and the optimal dose range was 65.5~81.3 mg/kg.

**Table 2:** Effects of dietary ZnVal supplementation on growth performance in piglets

Item	ZnVal (mg/kg)					SEM	*P* value		
	0	25	50	75	100		ANOOVO	Linear	Quadratic
Initial BW, kg	10.69	10.70	10.69	10.68	10.70	0.11	1.00	0.94	0.99
D14 BW, kg	15.78	15.88	16.10	16.45	16.17	0.15	0.67	0.84	0.62
D28 BW, kg	23.02	23.73	24.12	24.49	24.14	0.19	0.13	0.26	0.17
Days 1 to 14									
ADG, g	369.26	369.49	386.38	412.28	390.55	5.66	0.09	1.00	0.46
ADFI, g	605.95	604.24	613.91	624.70	612.50	10.24	0.93	0.92	0.83
FCR	1.64	1.64	1.60	1.52	1.58	0.02	0.37	0.93	0.62
Days 15 to 28									
ADG, g	517.26^b^	561.02^ab^	565.68^ab^	572.34^a^	568.97^a^	5.66	0.01	0.02	0.048
ADFI, g	881.40	912.43	933.85	934.08	936.31	13.62	0.43	0.38	0.51
FCR	1.71	1.63	1.65	1.65	1.66	0.02	0.87	0.43	0.49
Days 1 to 28									
ADG, g	440.34^b^	465.25^ab^	478.14^a^	491.68^a^	479.76^a^	3.98	<0.01	0.05	0.02
ADFI, g	743.68	758.33	773.88	779.39	774.41	10.81	0.57	0.62	0.61
FCR	1.69	1.63	1.62	1.60	1.62	0.01	0.20	0.21	0.17

^1^*BW* body weight, *ADG* average daily gain, *ADFI* average daily feed intake, *FCR* feed conversion ratio

^2^*SEM* standard error of the mean (*n* = 6)

a,b Values within a row with different superscripts differ significantly at *P* < 0.05

### Diarrhea Rate

As shown in Table 3, the supplementation of 25 to 100 mg/kg ZnVal significantly reduced (*P* < 0.01) the diarrhea rate of weaned piglets at 1 to 14 days and 1 to 28 days.

**Table 3:** Effects of dietary ZnVal supplementation on diarrhea rate (%) in piglets

Item	ZnVal (mg/kg)					SEM^1^	*P* value
	0	25	50	75	100		
Days 1 to 14	8.33^a^	4.17^b^	2.98^c^	3.13^c^	5.65^b^	0.99	<0.01
Days 15 to 28	3.87	2.53	2.08	1.93	2.68	0.34	0.19
Days 1 to 28	6.10^a^	3.35^b^	2.53^c^	2.53^c^	4.17^b^	0.66	<0.01

^1^*SEM* standard error of the mean (*n* = 6)

a,b Values within a row with different superscripts differ significantly at *P* < 0.05

### Antioxidant Capacity in Serum

Table 4 shows the effects of dietary ZnVal supplementation on the activity of antioxidant enzymes in the serum of piglets. There was a quadratic (*P* < 0.05) increase in serum Cu/Zn-SOD and a decrease in MDA activities with 25–100 mg/kg ZnVal supplementation on days 14 and 28. The supplementation of ZnVal increased (linear and quadratic, *P* < 0.05) GSH-Px activity in serum on day 14. However, no treatment differences were detected in the activity of SOD and T-AOC on day 14 or 28 in serum (*P* > 0.05). According to the quadratic fit model of dietary ZnVal supplementation level, the optimal supplemental level of ZnVal for piglets based on Cu/Zn-SOD activity (*Y* = −1.137 × 10^−3^*X*^2^ + 0.1723*X* + 25.74, *R*^2^ = 0.78) from days 1~14 was 75.8 mg/kg and the optimal dose range was 70.7~80.9 mg/kg.

**Table 4:** Effects of ZnVal supplementation on serum antioxidant capacity in piglets

Item^1^	ZnVal (mg/kg)					SEM^2^	*P* value		
	0	25	50	75	100		ANOVO	Linear	Quadratic
Day 14									
SOD, U/mL	53.55	53.65	50.51	51.60	47.09	1.26	0.47	0.24	0.64
GSH-Px, μmol/L	21.55^b^	26.65^a^	27.49^a^	28.17^a^	27.35^a^	0.66	<0.01	<0.01	0.01
MDA, nmol/mL	1.67^a^	1.50^b^	1.48^b^	1.52^b^	1.52^b^	0.02	<0.01	<0.01	<0.01
T-AOC, U/mL	8.88	9.11	9.44	9.56	9.58	0.13	0.36	0.31	0.56
Cu/Zn-SOD, U/mL	26.17^c^	28.94^b^	30.11^b^	34.53^a^	30.70^b^	0.56	<0.01	0.07	<0.01
Day 28									
SOD, U/mL	50.34	48.71	50.08	53.29	42.38	1.39	0.14	0.04	0.14
GSH-Px, μmol/L	27.06	29.10	28.80	29.23	28.87	0.56	0.79	0.38	0.41
MDA, nmol/mL	1.68^a^	1.54^b^	1.50^b^	1.50^b^	1.49^b^	0.01	<0.01	<0.01	0.01
T-AOC, U/mL	8.95	9.40	9.26	9.75	9.28	0.16	0.67	0.40	0.40
Cu/Zn-SOD, U/mL	27.11^b^	31.68^a^	32.86^a^	34.39^a^	32.99^a^	0.62	<0.01	<0.01	<0.01

^1^*SOD* superoxide dismutase, *GSH-Px* glutathione peroxidase, *MDA* malondialdehyde, *T-AOC* total antioxidant capacity, *Cu/Zn-SOD* copper/zinc-superoxide dismutase

^2.^*SEM*, standard error of the mean (*n* = 6)

^a,b,c^Values within a row with different superscripts differ significantly at *P* < 0.05

### Antioxidant Capacity in Liver

Table 5 shows the effects of dietary ZnVal supplementation on the activity of antioxidant enzymes in the liver of piglets. The supplementation of 75 mg/kg ZnVal increased (*P* < 0.05) SOD activity in the liver and dietary with 25–100 mg/kg ZnVal supplementation increased the Cu/Zn-SOD and T-AOC activities in the liver (linear and quadratic, *P* < 0.05) as compared to the CON group.

**Table 5:** Effects of ZnVal supplementation on liver antioxidant capacity in piglets

Item^1^	ZnVal (mg/kg)					SEM^2^	*P* value		
	0	25	50	75	100		ANOVO	Linear	Quadratic
SOD, U/mg	53.19^b^	50.53^b^	54.83^ab^	74.54^a^	51.34^b^	2.65	<0.01	0.21	0.18
GSH-Px, μmol/g	24.65	25.03	25.78	28.50	25.28	0.54	0.16	0.21	0.24
MDA, nmol/ mg	0.80	0.90	0.92	0.65	0.79	0.04	0.20	0.34	0.52
T-AOC, U/mg	2.64^b^	5.38^a^	6.41^a^	5.29^a^	5.00^a^	0.31	<0.01	<0.01	<0.01
Cu/Zn-SOD, U/mg	10.17^b^	19.85^a^	20.57^a^	19.56^a^	18.73^a^	1.02	<0.01	<0.01	<0.01

^1^*SOD* superoxide dismutase, *GSH-Px* glutathione peroxidase, *MDA* malondialdehyde, *T-AOC* total antioxidant capacity, *Cu/Zn-SOD* copper/zinc-superoxide dismutase

^2^*SEM* standard error of the mean (*n* = 6)

^a,b^Values within a row with different superscripts differ significantly at *P* < 0.05

### Immunity Status in Serum

As shown in Table 6, higher (linear, *P* < 0.05) concentrations of IgG in the serum were observed in piglets supplemented with 75–100 mg/kg ZnVal on day 14 and dietary supplementation with 25–100 mg/kg ZnVal increased (linear and quadratic, *P* < 0.05) the level of IgG in serum on day 28 compared with the CON group. However, there were no significant differences in the serum concentration of IgA and IgM among all groups on day 14 or 28 (*P* > 0.05).

**Table 6:** Effects of ZnVal supplementation on serum immunity status in piglets

Item^1^	ZnVal(mg/kg)					SEM^2^	*P* value		
	0	25	50	75	100		ANOVO	Linear	Quadratic
Day 14									
IgG (mg/mL)	7.82^b^	8.67^ab^	8.71^ab^	8.93^a^	8.89^a^	0.12	0.01	<0.01	0.07
IgA (μg/mL)	14.73	14.65	15.10	14.33	13.14	0.42	0.65	0.36	0.36
IgM (μg/mL)	6.52	6.22	6.16	6.72	6.28	0.16	0.82	0.42	0.81
Day 28									
IgG (mg/mL)	7.47^b^	8.93^a^	8.73^a^	9.34^a^	8.68^a^	0.16	<0.01	<0.01	<0.01
IgA (μg/mL)	14.74	15.12	15.03	15.46	15.84	0.26	0.74	0.34	0.82
IgM (μg/mL)	5.20	6.11	6.14	6.25	6.06	0.17	0.30	0.19	0.14

^1^*IgG* immunoglobulin G, *IgA* immunoglobulin A, *IgM*, immunoglobulin M

^2^*SEM* standard error of the mean (*n* = 6)

^a,b^Values within a row with different superscripts differ significantly at *P* < 0.05

### Immunity Status in Small Intestine

As shown in Table 7, higher (*P* < 0.05) concentrations of IgA in the duodenum and ileum were observed in piglets supplemented with 75 mg/kg ZnVal compared to other groups and the supplementation of 25–100 mg/kg ZnVal showed a higher (linear and quadratic, *P* < 0.05) concentration of IgG in duodenum as compared to the CON group, while did not affect IgM concentration (*P* > 0.05).

**Table 7:** Effects of dietary ZnVal supplementation on small intestinal immunity status in piglets

Item^1^	ZnVal (mg/kg)					SEM^2^	*P* value		
	0	25	50	75	100		ANOVO	Linear	Quadratic
Duodenum									
IgG (mg/mg)	7.35^b^	10.74^a^	10.89^a^	10.90^a^	10.81^a^	0.39	<0.01	0.01	<0.01
IgA (μg/mg)	10.26^b^	10.64^b^	10.61^b^	17.68^a^	11.05^b^	0.72	<0.01	0.15	0.13
IgM (μg/mg)	6.27	6.64	6.68	6.61	6.39	0.20	0.96	0.86	0.47
Jejunum									
IgG (mg/mg)	9.26	10.07	9.65	10.15	9.89	0.40	0.96	0.70	0.73
IgA (μg/mg)	10.23	10.43	10.93	13.26	10.80	0.45	0.20	0.58	0.34
IgM (μg/mg)	5.77	6.08	6.11	6.17	6.07	0.23	0.99	0.77	0.71
Ileum									
IgG (mg/mg)	6.86	6.99	6.99	7.78	6.94	0.19	0.54	0.64	0.48
IgA (μg/mg)	12.32^b^	14.20^ab^	14.23^ab^	14.74^a^	14.29^ab^	0.26	0.02	0.04	0.03
IgM (μg/mg)	4.92	5.19	5.23	5.42	5.20	0.14	0.87	0.74	0.50

^1^*IgG* immunoglobulin G, *IgA* immunoglobulin A, *IgM* immunoglobulin M

^2^*SEM* standard error of the mean (*n* = 6)

^a,b^Values within a row with different superscripts differ significantly at *P* < 0.05

### Intestinal Morphology

As shown in Table 8, dietary supplementation with 50–100 mg/kg ZnVal increased the villus height and villus height/crypt depth of jejunum compared with the CON group (linear, *P* < 0.05). However, ZnVal did not affect the morphology of the duodenum and ileum.

**Table 8:** Effects of dietary ZnVal supplementation on small intestinal morphology in piglets

Item^1^	ZnVal(mg/kg)					SEM^1^	*P* value		
	0	25	50	75	100		ANOVO	Linear	Quadratic
Duodenum									
VH, μm	328.78	361.70	364.50	372.19	363.42	7.58	0.26	0.04	0.47
CD, μm	506.04	527.31	503.73	502.23	508.63	9.27	0.68	0.75	0.92
VH/CD	0.65	0.69	0.72	0.75	0.75	0.01	0.08	<0.01	0.43
Jejunum									
VH, μm	338.48^c^	379.44^bc^	398.76^ab^	434.94^a^	432.62^ab^	8.60	<0.01	<0.01	0.17
CD, μm	323.21	337.47	325.12	351.61	340.24	5.67	0.52	0.25	0.8
VH/CD	1.05^c^	1.13^bc^	1.23^ab^	1.24^ab^	1.27^a^	0.02	<0.01	<0.01	0.10
Ileum									
VH, μm	334.24	346.21	336.01	355.35	349.21	6.03	0.80	0.64	0.90
CD, μm	293.32	302.48	304.04	306.81	295.00	4.33	0.85	0.81	0.29
VH/CD	1.14	1.15	1.10	1.12	1.18	0.01	0.27	0.18	0.08

^1^*VH* villus height, *CD* crypt depth, *VH/CD* villus height/crypt depth ratio

^2^*SEM* standard error of the mean (*n* = 6)

^a,b,c^Values within a row with different superscripts differ significantly at *P* < 0.05

### Zinc Content of Tissues

Table 9 shows the effects of dietary ZnVal supplementation on the zinc concentration of tissues in piglets. Dietary supplementation with 75–100 mg/kg ZnVal showed a higher (linear, *P* < 0.05) concentration of zinc in the liver compared to other groups. Besides, higher (linear and quadratic, *P* < 0.05) concentrations of zinc in the heart, spleen, and kidney were observed in piglets supplemented with 50–100 mg/kg ZnVal compared to those in piglets supplemented with the CON diet.

**Table 9:** Effects of dietary ZnVal supplementation on zinc concentration of tissues in piglets

Item	ZnVal (mg/kg)					SEM^1^	*P* value		
	0	25	50	75	100		ANOVO	Linear	Quadratic
Heart (mg/kg)	15.87^b^	17.59^ab^	18.76^a^	18.13^a^	18.08^a^	0.28	<0.01	<0.01	<0.01
Liver (mg/kg)	64.42^b^	68.55^b^	64.32^b^	88.76^a^	89.65^a^	2.79	<0.01	<0.01	0.18
Spleen (mg/kg)	19.35^b^	20.09^ab^	21.99^a^	21.48^a^	21.78^a^	0.27	<0.01	<0.01	0.08
Kidney (mg/kg)	19.24^b^	20.00^b^	22.37^a^	22.38^a^	22.41^a^	0.32	<0.01	<0.01	0.02

^1^*SEM* standard error of the mean (*n* = 6)

^a,b^Values within a row with different superscripts differ significantly at *P* < 0.05

## Discussion

Zinc is a component of various enzymes in animals; it has important physiological and nutritional functions for animal growth, reproduction, and immunity, as well as exhibits cell growth promotion functions [18]. As a new efficient green feed additive, dietary supplementation of ZnMet and ZnGly was shown to improve growth performance [9, 12]. The improvement in growth performance may be related to the promotion of rapid proliferation of taste bud cells in tongue mucosa by zinc, thus prolonging the residence time of the feed in the digestive tract, improving the secretion of the digestive system, and increasing the activity of enzymes in tissue cells [19, 20]. In addition, the supplementation of 75 mg/kg zinc methionine hydroxy analog chelate increased the laying rate and egg weight and decreased the FCR of aged broiler breeders [21]. In the present study, we demonstrated that the supplementation of 75–100 mg/kg ZnVal improved ADG on days 15–28 and dietary 50–100 mg/kg ZnVal supplementation increased ADG on days 1–28 compared with CON, but had no effect on final BW, ADFI, and FCR of weaned piglets. This finding is in agreement with Wang et al. [22], who reported 100 mg/kg glycine zinc improves ADG, but did not affect ADFI and FCR of weaned piglets. Contrary to the present study, Liu et al. [23], Li et al. [24], and Xie et al. [25] reported that ZnMet had no effect on ADG, ADFI, and FCR in weaned piglets. The discrepancies may be attributed to variations in the bioavailability of diverse amino acid chelated zinc and differences in the types of diets consumed.

Zinc oxide (ZnO) has been used as an antibacterial agent in conventional monogastric breeding zootechnical systems for many years [26]. It has been commonly used during the weaning of piglets, which is characterized by oxidative stress, barrier dysfunction, and intestinal microflora disturbance [27]. However, the high consumption of Zn by pigs leads to the excretion of a considerable amount of Zn in urine and feces, which raises concerns about environmental pollution and causes a negative public perception of ZnO [28, 29]. In this study, the addition of 25–100 mg/kg ZnVal significantly reduced the diarrhea rate in piglets on days 1–14 and 1–28, which contrasts with the findings of Diao [7], who reported no significant effect on diarrhea rate in piglets supplemented with 100 mg/kg ZnGly. Our findings suggest that the reduction of diarrhea rate in our study is indicative of improved intestinal health in piglets. Moreover, adequate levels are required to maintain the gut barrier, avoid risk intestinal infections, and prevent diarrhea and zinc deficiency can alter the paracellular ionic conductance, and perturb barrier integrity, thereby reducing Cl^−^ secretion and increasing susceptibility to infection [30].

Malonaldehyde is a significant biomarker for assessing the level of oxidative stress in weaned piglets and this compound is primarily generated through the process of lipid peroxidation [31]. Antioxidant enzymes, specifically SOD and GSH-Px, are crucial in the metabolism and detoxification of reactive oxygen species. The function and structure of Cu/Zn-SOD, which represents 90% of total SOD concentration, are dependent on the availability of zinc. Therefore, Cu/Zn-SOD can be used as a biomarker to evaluate the zinc status in the body [32, 33]. The greater serum GSH-Px, Cu/Zn-SOD, and T-AOC activities along with lower MDA concentration indicate that ZnVal may decrease the occurrence of lipid peroxidation and enhance the antioxidant capacity. Previous studies demonstrated that 100 mg/kg ZnGly increased Cu/Zn-SOD activity in serum in piglets and 60 mg/kg ZnMet increased T-AOC, GSH-Px activity in serum, and T-AOC, Cu/Zn-SOD, and GSH-Px activities in the liver of laying hens [9, 22]. In addition, Zhu et al. [12] demonstrated that 60 mg/kg ZnGly reduced MDA content and increased T-SOD and T-AOC activities in serum in broilers. Zinc could regulate the synthesis of antioxidant proteins, and it is reported that zinc affects Nrf2 expression by activating the AKT/GSK-3β signaling pathway and reducing Nrf2 trafficking and Fyn protein degradation [34]. Additionally, zinc regulates GSH synthesis via Nrf2 [35]. Furthermore, zinc inhibits the activity of reduced nicotinamide adenine dinucleotide phosphate (NADPH) oxidase, which reduces the production of free radicals. NADPH is vital for glutathione production and maintenance of glutathione reductase activity [36].

Immunoglobulin G, Immunoglobulin M, and Immunoglobulin A are the main immunoglobulins produced by activated B lymphocytes, which reflect the humoral immune status of the body. The current study indicated that dietary supplementation with 25–100 mg/kg ZnVal increased the level of IgG in serum on day 28. Higher concentration of IgG in serum was observed in piglets supplemented with 75–100 mg/kg ZnVal on day 14. In addition, higher concentrations of IgA in the duodenum and ileum were observed in piglets supplemented with 75 mg/kg ZnVal and the supplementation of 25–100 mg/kg ZnVal showed a higher concentration of IgG in the duodenum, indicating that dietary supplementation with ZnVal can improve intestinal immune function in weaned piglets. Previous studies demonstrated that 50 mg/kg ZnMet can increase concentrations of serum IgA and 120 mg/kg ZnMet resulted in higher serum content of IgG in piglets [24, 25]. Additionally, Levkut et al. [37] reported that 30 mg/kg ZnGly upregulated the expression of IgA genes in the broiler small intestine and increased the concentration of sIgA in the brush border.

The intestinal tract is the largest organ in the immune system of animals, and maintaining normal intestinal barrier function is important for good intestinal health. Piglets often face significant changes in intestinal structure and function after weaning, mainly manifested by villi atrophy and crypt hyperplasia that leads to a decrease in the ability to absorb nutrition as the small intestine villi are the important site for nutrient absorption [38]. Villus height, crypt depth, and the ratio of villus height to crypt depth are commonly used to evaluate intestinal function. Longer villi provide more areas for the absorption of nutrients, while deeper crypts indicate renewal of intestinal epithelial cells. In this study, dietary supplementation with 50–100 mg/kg ZnVal increased villus height and the ratio of villus height to crypt depth of jejunum, which can partially explain the observed improvement in ADG. This result was partially consistent with Diao et al. [7], who observed that 100 mg/kg ZnVal increased villus height and the ratio of villus height to crypt depth of the jejunum in piglets. Similarly, Zhu et al. [12] found that 60 mg/kg ZnGly significantly increased the villus height in the duodenum and jejunum and decreased crypt depth in the duodenum in broilers. In addition, Li et al. [39] reported that 80 mg/kg ZnMet increased villus height, villus area, and villus height/crypt depth ratio but reduced crypt depth in the jejunum in laying hens. Zinc has been shown to promote cell differentiation through the PI3K/AKT/mTOR signaling pathway and upregulate the expression of tight junction protein zonula occludens-1 (ZO-1), consequently enhancing the barrier function of the intestinal mucosa [40]. Recombinant Mucin 2 (MUC2) is predominantly secreted by goblet cells, composing the bulk of the intestinal mucus. MUC2 serves key biological functions, lubricating the intestinal tract and facilitating the adhesion of intestinal antibacterial proteins and symbiotic flora. Moreover, it helps prevent the infiltration of harmful pathogens and substances into the intestinal tract. Levkut et al. [37] showed that supplementation of 30 mg/kg zinc glycinate and zinc sulfate in broiler diets both upregulated the expression of the MUC2 gene in the jejunum. Therefore, dietary supplementation with ZnVal could enhance the function of the intestinal barrier and promote intestinal health.

Amino acid chelated zinc has been reported to have higher bioavailability compared to other sources of zinc. Thus, to evaluate zinc absorption and utilization, we measured the concentration of zinc in tissues, which also serves as an indicator of the body’s nutritional status [41]. Besides, the liver contains a relatively high and stable concentration of trace elements, which reasonably represents the deposition of these elements within the body. Upon blood absorption, approximately 67–80% of zinc accumulates in the liver, spleen, and kidneys; comparatively smaller amounts are detected in muscles and other tissues [42]. Therefore, each tissue exhibits diverse capacities for zinc accumulation, leading to distinct concentrations. In this study, the supplementation of 75–100 mg/kg ZnVal showed a higher concentration of zinc in the liver and dietary supplementation with 50–100 mg/kg ZnVal increased the concentrations of zinc in the heart, spleen, and kidney, which indicated that the addition of ZnVal in diet improved the absorption of trace elements. These results were partly in accordance with the results of Liu et al. [23], who reported that dietary supplementation with 150 mg/kg ZnMet showed a higher concentration of zinc in the liver in piglets. Furthermore, Zhang et al. [43] reported that the supplementation of ZnMet to growing-finishing pigs significantly increased the concentrations of zinc in the muscle, liver, kidney, and serum in comparison with the ZnSO_4_ supplementation. In addition, Jahanian et al. [19] found that incremental levels of ZnMet increased zinc concentrations in the liver and thymus of broiler chicks. Therefore, the addition of ZnVal showed a higher bioavailability, which is consistent with the results of growth performance.

## Conclusion

In conclusion, the present research showed that supplementation of ZnVal improves growth performance by increasing antioxidant capacity and immunity and regulating intestinal morphology and the optimal inclusion level of ZnVal was 65~80 mg/kg.

## Data Availability

The data used to support the findings are all included in the article.

## Abbreviations

ZnVal: Valine chelated zinc
ADG: Average daily gain
ADFI: Average daily feed intake
FCR: Feed conversion rate
T-AOC: Total antioxidant capacity
SOD: Superoxide dismutase
MDA: Malonaldehyde
GSH-Px: Glutathione peroxidase
Cu/Zn-SOD: Copper/zinc-superoxide dismutase
IgA: Immunoglobulin A
IgG: Immunoglobulin G
IgM: Immunoglobulin M
